# Long non-coding RNA PRNCR1 modulates non-small cell lung cancer cell proliferation, apoptosis, migration, invasion, and EMT through PRNCR1/miR-126-5p/MTDH axis

**DOI:** 10.1042/BSR20193153

**Published:** 2020-07-23

**Authors:** Ran Guo, Tongchen Hu, Yanheng Liu, Yinzai He, Yanling Cao

**Affiliations:** 1 Department of Thoracic Neoplasms Surgery, Inner Mongolia People’s Hospital, Inner Mongolian, Hohhot 010010, China; 2 Department of Cardiothoracic Surgery, The People’s Hospital of Leshan, Leshan, Sichuan 614000, China; 3 Department of Rhumatic, Inner Mongolia People’s Hospital, Inner Mongolian, Hohhot 010010, China

**Keywords:** metadherin (MTDH), miR-126-5p, Non-small cell lung cancer (NSCLC), prostate cancer non-coding RNA 1 (PRNCR1)

## Abstract

**Background:** Non-small cell lung cancer (NSCLC) is a highly malignant tumor. Accumulating evidence suggested that prostate cancer non-coding RNA 1 (PRNCR1) participated in the pathogenesis of NSCLC, whereas the elaborate mechanism remains unclear. Hence, the role of PRNCR1 in the progression of NSCLC was investigated.

**Methods:** Levels of PRNCR1, microRNA-126-5p (miR-126-5p), and metadherin (MTDH) were examined by quantitative real-time polymerase chain reaction (qRT-PCR). Cell proliferation was measured using Cell Counting Kit-8 (CCK-8). Flow cytometry was conducted to determine cell apoptosis. Besides, transwell assay was performed to detect cell migration and invasion in NSCLC cells. The expression levels of E-cadherin, N-cadherin, Vimentin, and MTDH were detected via Western blot. Dual-luciferase reporter, RNA immunoprecipitation, and RNA pull down assays were employed to verify the relationship between miR-126-5p and PRNCR1 or MTDH.

**Results:** PRNCR1 and MTDH levels were highly expressed, while miR-126-5p expression was lowly expressed in NSCLC tissues and cell lines. Knockdown of PRNCR1 promoted cell apoptosis, impeded proliferation, migration, invasion, and epithelial-mesenchymal transition (EMT) in NSCLC cells, and these effects were abrogated by its target gene of miR-126-5p inhibitor. Moreover, MTDH as the target of PRNCR1, its overexpression reversed the impacts of miR-126-5p mimic on cell behaviors and EMT *in vitro*. Finally, PRNCR1 and miR-126-5p regulated MTDH expression.

**Conclusion:** PRNCR1 modified cell behaviors and EMT via miR-126-5p/MTDH axis in NSCLC cells, providing a novel thinking for clinical treatment of NSCLC.

## Introduction

Lung cancer is the respiratory tract cancer with high malignancy [1], of which non-small cell lung cancer (NSCLC) is the significant member and approximately occupies 85% of lung cancer [2], while the 5-year survival rate of NSCLC patients is relatively low [3]. Given that the clinical features of NSCLC, early-stage of NSCLC was mostly out of sighted and nonspecific. Recently, platinum combined with other chemotherapeutic drugs, such as taxanes, vinorelbine gemcitabine, or pemetrexed, are the major operations for treating advanced NSCLC [4]. Despite the advance of the therapeutic techniques in diagnosis and surgery, the 5-year survival rate of human NSCLC patients was not significantly improved [5]. Besides, NSCLC cells with the high metastasis capability could escape the normal mediation of apoptosis and division, which directly causes the failure of NSCLC treatment. Hence, it is expected to find the effective therapeutic methods via curbing cell proliferation and metastasis in NSCLC.

The long non-coding RNAs (lncRNAs), were a type of non-coding RNAs (ncRNAs) with the length over than 200 bp, have been reported to characterize the progression and initiation of tumors via epigenetic, transcriptional, and post-transcriptional modulations [6]. For instance, metastasis-associated lung adenocarcinoma transcript 1 (MALAT1) was overexpressed in NSCLC [7]. It was additionally demonstrated that MALAT1 might accelerate epithelial-mesenchymal transition (EMT) development of NSCLC [8], and metastasis of lung cancer *in vitro* [9]. Furthermore, prostate cancer non-coding RNA 1 (PRNCR1), which is transcribed from 8q24 region [10], has been displayed as a deciphered gene of human prostate cancer [11] and is an oncogene in various diseases, such as colorectal cancer [12] and NSCLC [13]. Nevertheless, the role of PRNCR1 in the pathogenesis and tumorigenesis of NSCLC remains unclear. This investigation was aimed to uncover the functional mechanism of PRNCR1 in the progression and initiation of NSCLC.

Accruing evidence suggested that ncRNAs may be grimly accountable for regulating downstream gene expression in human [14,15]. Of which microRNAs (miRNAs) are a class of ∼22 nucleotides (nt), belong to ncRNAs. Bartel et al. [16] revealed that miRNAs modulated gene expression via targeting downstream gene mRNAs for cleavage or translational suppression. Among them, miR-126-5p is an intronic miRNA, and it was proved as a tumor-inhibitory factor in multiple human tumors, including prostate cancer [17], breast cancer [18], and NSCLC [19]. Nevertheless, the impact of miR-126-5p on the pathogenesis of human tumors, including NSCLC, was not completely understood.

Metadherin (MTDH), which is also named as astrocyte-elevated gene-1 protein (AEG-1), was an up-regulated gene in breast cancer. In the meanwhile, MTDH has been uncovered to contribute to cell proliferation and chemoresistance of tumors via activating Forkhead box O3 [20]. The high expression of MTDH was closely related to the survival rate of breast cancer patients [21]. In the research, MTDH was considered to be an oncogene in NSCLC and then this hypothesis was confirmed in the subsequent assays.

In the present study, we focused on the expression of PRNCR1, miR-126-5p, and MTDH1 in NSCLC tissues and cell lines, the functional mechanisms of them were also investigated *in vitro*. Additionally, the study also proved the regulatory mechanism between PRNCR1 and miR-126-5p or MTDH1.

## Materials and methods

### Clinical samples and cell culture

Forty samples of NSCLC and the paired para-carcinoma tissues (*n*=40) were obtained at Inner Mongolia People’s Hospital. Prior to the study, it was approved by the Ethics Committee of Inner Mongolia People’s Hospital. Each participator signed the written informed consent prior to surgery operation and did not undergo any other treatments, such as chemotherapy and radiotherapy within three months. When the human tissue samples were received, they were immediately maintained at −80°C.

Besides, NSCLC cell lines, such as SPC-A1 and A549, and control of Normal human lung epithelial cells, BEAS-2B were purchased from Be Na culture collection (Beijing, China). According to the description of the company, SPC-A1 and A549 were cultured with Roswell Park Memorial Institute (RPMI)-1640 (HyClone, Logan, UT, U.S.A.), and control cells of BEAS-2B were incubated using Dulbecco’s modified Eagle’s medium (DMEM; HyClone). Complete medium of RPMI-1640 and DMEM was compounded with supplementing 10% fetal bovine serum (FBS; Thermo Fisher Scientific, Rockford, IL, U.S.A.) and Penicillin (100 U/ml, Gibco, Carlsbad, CA, U.S.A.)-Streptomycin (100 µg/ml, Gibco). Finally, cells were cultured at 37°C with 5% CO_2_ atmosphere.

### qRT-PCR

Briefly, total RNA was separated and extracted from NSCLC tissues and cell lines by using TRIzol (Invitrogen, Carlsbad, CA, U.S.A.) in line with the manufacturer’s manuals. RNA was converted to the first-strand cDNA via Reverse Transcription System (Promega, Madison, WI, U.S.A.) based on the producer’s protocols. Then, qPCR was carried out utilizing Premix Ex Taq™ (Perfect Real Time) (Takara, Dalian, China) with cDNA as template, and placed on the Roche LC480 system (Roche Applied Science, Mannheim, Germany). The specific primers for qPCR in the study were obtained in Genechem (Shanghai, China), and the sequences were as follows: PRNCR1 (forward, 5′-CCAGATTCCAAGGGCTGATA-3′ and reverse, 5′-GATGTTTGGAGGCATCTGGT-3′); miR-126-5p (forward, 5′-GGTATAATCCGCCGCTTAGCTGCC-3′ and reverse, 5′-GTGCAGGGTTGCAAGGT-3′); MTDH (forward, 5′-AGCCCAAACCAAATGGACG-3′ and reverse, 5′-AACTGTTTTGCACTGCTTTAGC-3′); β-actin (forward, 5′-CCAGATTCCAAGGGCTGATA-3′ and reverse, 5′-GATGTTTGGAGGCATCTGGT-3′); and U6 (forward, 5′-CTCGCTTCGGCAGCACA-3′, and reverse, 5′-AACGCTTCACGAATTTGCGT-3′). From the above sequences, the relative levels of PRNCR1, miR-126-5p, and MTDH were determined with β-actin and U6 as internal references via 2^−ΔΔ*C*_t_^ method.

### Transient transfection

Small interfering RNA (siRNA) against PRNCR1 (si-PRNCR1) was used to silence PRNCR1, and siRNA scramble functioned as its control (si-NC), overexpression of PRNCR1 (pcDNA-PRNCR1) and MTDH (MTDH) were applied to upregulate levels of PRNCR1 and MTDH, pcDNA3.1 empty vector (pcDNA-NC or vector) acted as their control. In addition, oligonucleotides of miR-126-5p mimic (miR-126-5p) and inhibitor (anti-miR-126-5p), as well as the relative controls (NC or anti-NC) were also applied in the present study. All vectors and oligonucleotides were synthesized from Genechem, and transfection was carried out utilizing Lipofectamine™ 2000 (Invitrogen) following producer’s instructions.

### CCK-8

Cell proliferation was carried out using the reagent kit of cell counting kit 8 (CCK-8) (Solarbio, Beijing, China). The transfected cells (SPC-A1 and A549) were uniformly plated in the bottom well of a 96-well plate and incubated with complete RPMI-1640 medium for 0, 24, 48, and 72 h, severally. Subsequently, CCK-8 solution with 10-µl volume was added into per well of 96-well plate, then cultured for another 2 h at 37°C. Absorbance was detected using the multi-well microplate reader (Bio-Rad, Hercules, CA, U.S.A.) at 450 nm.

### Flow cytometry

Transfected cells of SPC-A1 and A549 were harvested and re-suspended with binding buffer at the density of 1 × 10^6^ cells/ml. Subsequently, Annexin V-fluorescein isothiocyanate/propidium iodide (Annexin V-FITC/PI; Sigma, St. Louis, MO, U.S.A.) was performed to stain cells according to manufacturer’s introductions. Cell apoptotic rate was analyzed via a FACSCalibur flow cytometer (BD Biosciences, San Jose, CA, U.S.A.).

### Transwell assay

First, 4 × 10^4^ cells were suspended in the medium under serum-free condition and then cells were seeded into the upper chamber (8-µm pore size; Corning, Corning, NY, U.S.A.) with uncoated (cell migration assay) or Matrigel-coated (cell invasion assay) membrane, and then medium containing 10% FBS was added into the lower chamber. Afterward, cells remaining on the upper membrane were lightly erased with cotton swabs, and the rest of the cells were fixed with 4% paraformaldehyde (PFA) at 24 h post-incubation. Then, cells were darkly stained by 0.1% crystal violet for 20 min. In the endpoint, cells were counted and imaged with an inverted microscope (Olympus, Tokyo, Japan).

### Western blot

Total protein was harvested and extracted from NSCLC tissue samples and cell lines (SPC-A1 and A549) with RIPA buffer (Millipore, Bedford, MA, U.S.A.) and the protein concentration was calculated with reagent kit via Bradford method (Beyotime, Shanghai, China). Then, protein lysates were isolated via 12% sodium dodecyl sulfate/polyacrylamide gel electrophoresis (SDS/PAGE), and then transfected onto polyvinylidene fluoride (0.45 µm, PVDF) membranes (Millipore). The membranes were blocked with 5% bovine serum albumin (BSA; Absin Bioscience, Shanghai, China) in 1× Tris-buffered Saline Tween-20 (TBST; Absin Bioscience), and then incubated with specific diluted primary antibodies, including E-cadherin (1:1000, ab76055), Vimentin (1:1000, ab8979), N-cadherin (1:500, ab18203), and MTDH (1:800, ab45338), as well as glyceraldehyde-3-phosphate dehydrogenase (GAPDH; 1:5000, ab8245) as endogenous control, and all primary antibodies were purchased from Abcam (Cambridge, MA, U.S.A.). After the membranes were incubated overnight at 4°C, 1× TBST was used to wash membranes for thrice, followed by covering the corresponding second antibody. Finally, protein bands were visualized using ECL Reagent (Millipore).

### Dual-luciferase reporter assay

The common fragments of miR-126-5p and PRNCR1 (wild-type and mutant) were inserted into pmirGLO (Promega) to construct wild-type (PRNCR1-wt) and mutant (PRNCR1-mut) luciferase reporter vectors of PRNCR1. Additionally, the potential binding domain between miR-126-5p and wild-type or mutant of MTDH were introduced into pmirGLO, and then they formed luciferase reporter vectors of MTDH-wt and MTDH-mut. These reporter vectors were co-transfected with Renilla vector (pRL-CMV, Promega), and miR-126-5p mimic or inhibitor was transfected into SPC-A1 and A549 cells using Lipofectamine™ 2000 (Invitrogen), respectively. After 48 h of transfection, cells were lysed, and the firefly and renilla luciferase activities were measured by using dual-luciferase reporter assay system (Promega) via a Varioskan Flash (Thermo Fisher Scientific).

### RIP assay

NSCLC cells were transfected with NC, miR-126-5p, PRNCR1, or control, respectively. After transfection for 48 h, Magna RNA immunoprecipitation (RIP) RNA-Binding Protein Immunoprecipitation Kit (Millipore) with the transfected cells were employed to perform RIP assay. Based on the manuals, cells were incubated with anti-Ago2 antibody (Abcam) or negative control of IgG (Abcam). The relative abundance of PRNCR1, miR-126-5p, and MTDH was identified by quantitative real-time polymerase chain reaction (qRT-PCR).

### RNA pull-down assay

For RNA pull-down test, Bio-labeled probe of miR-126-5p (Bio-miR-126-5p) or its control (Bio-NC) was transfected into SPC-A1 and A549 cells. At 48 h post-incubation, cells were collected and lysed, and then Streptavidin-Dyna beads were incubated with lysates overnight at 4°C, along with RNA isolation. Finally, the enrichment of PRNCR1 was measured by qRT-PCR.

### Immunohistochemistry

The tumor samples were fixed with 4% (PFA; Solarbio), and embedded in paraffin. Multiple sections (5.0-µm thick) were cut from the paraffin-embedded NSCLC tissues. After that, the samples were incubated with primary antibody (MTDH, ab45338) overnight at 4°C. Next, the corresponding secondary antibody was selected to amplify the signals via incubation for 30 min at room temperature.

### Statistical analysis

The data were processed utilizing SPSS 22.0 and shown as the means ± standard deviation (SD). Data between two-group comparisons were analyzed using Student’s *t* test, and that of more than two group comparisons was analyzed via the one-way ANOVA method. All assays were performed in triplicate, and *P*-value less than 0.05 was considered statistically significant.

## Results

### Level of PRNCR1 was high, while miR-126-5p expression was low in NSCLC tissues and cell lines

**Figure 1:**
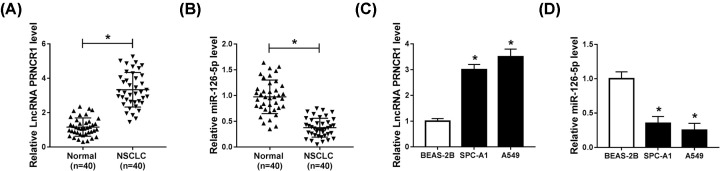
Level of PRNCR1 was high, while miR-126-5p expression was low in NSCLC tissues and cell lines (**A,B**) QRT-PCR was conducted to analyze PRNCR1 and miR-126-5p levels in paired NSCLC tissues and adjacent normal tissue samples. (**C,D**) Levels of PRNCR1 and miR-126-5p were determined by qRT-PCR in NSCLC cells (SPC-A1 and A549) and normal human lung epithelial cells (BEAS-2B). *P<0.05.

In the assay, we measured 80 samples, including 40 tumor samples and the paired adjacent normal tissues. The results of qRT-PCR exhibited that the expression of PRNCR1 was clearly augmented, while the level of miR-126-5p was evidently decreased in NSCLC tumors (Figure 1A,B). Synchronously, we selected two cell lines of SPC-A1 and A549 as the NSCLC cell model, and BEAS-1B cells acted as normal control. Then, the level of PRNCR1 and miR-126-5p was detected by qRT-PCR, and the results showed that there was the same tendency of PRNCR1 and miR-126-5p levels in NSCLC tissues and cell lines (Figure 1C,D). These data emerged that the level of PRNCR1 was increased, whereas miR-126-5p expression was impeded in NSCLC tissues and cell lines.

### Knockdown of PRNCR1 promoted cell apoptosis, impeded proliferation, migration, invasion, and EMT in NSCLC cells

**Figure 2:**
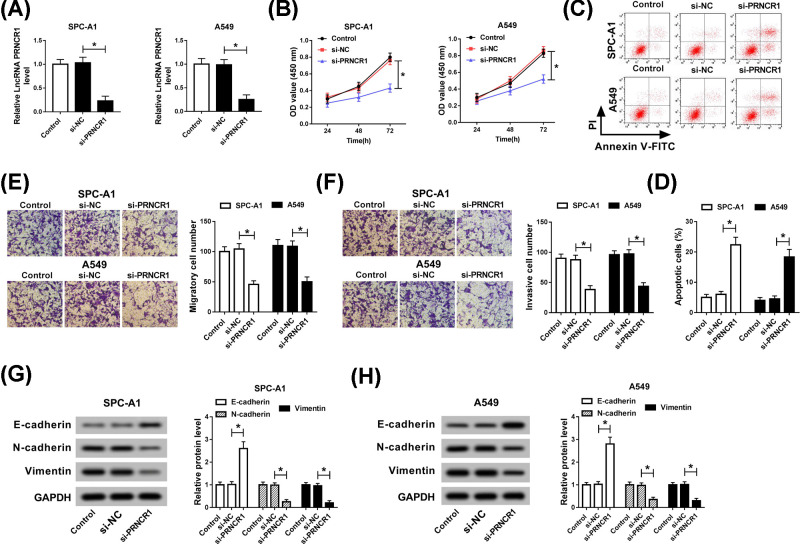
Knockdown of PRNCR1 promoted cell apoptosis, impeded proliferation, migration, invasion, and EMT in NSCLC cells SPC-A1 and A549 cells were transfected with si-PRNCR1 or si-NC, respectively. (**A**) qRT-PCR was applied to examine PRNCR1 expression in si-PRNCR1-treated SPC-A1 and A549 cells. (**B**) CCK-8 assay was employed to assess the role of si-PRNCR1 in SPC-A1 and A549 cells. (**C,D**) Cell apoptotic rate was determined via flow cytometry analysis. (**E,F**) Transwell assay was performed to evaluate migration and invasion of SPC-A1 and A549 cells. (**G,H**) Western blot assay was performed to examine the expression levels of EMT-relative proteins, including E-cadherin, N-cadherin, and Vimentin. *P<0.05.

In view of the aberrant expression of PRNCR1 in NSCLC tissues and cell lines, we guessed that PRNCR1 might participated in the progression of NSCLC. Subsequently, si-NC or si-PRNCR1 was introduced in SPC-A1 and A549 cells, and the knockdown efficiency of si-PRNCR1 was verified via qRT-PCR. The results of qRT-PCR demonstrated that the level of PRNCR1 was significantly restrained by si-PRNCR1 in SPC-A1 and A549 cells (Figure 2A). At the same time, cell proliferation was examined by CCK-8, and the results suggested that PRNCR1 silencing blocked the proliferation of SPC-A1 and A549 cells (Figure 2B). Furthermore, the apoptotic rate of NSCLC cells was obviously enhanced *in vitro* (Figure 2C,D). In the meanwhile, transwell assay was aimed to assess the capacities of cell migration and invasion, and the results discovered that knockdown of PRNCR1 could repress cell migration and invasion in both SPC-A1 and A549 cells (Figure 2E,F). Finally, because of E-cadherin, N-cadherin and Vimentin were the markers for EMT, the high expression of E-cadherin, and low expression of N-cadherin and Vimentin indicated that EMT was remarkably hindered by PRNCR1 deletion (Figure 2G,H). In brief, these findings confirmed that down-regulation of PRNCR1 notably promoted cell apoptosis, suppressed proliferation, migration, and invasion, as well as EMT in NSCLC cells.

### PRNCR1 functioned as a molecular sponge for miR-126-5p

**Figure 3:**
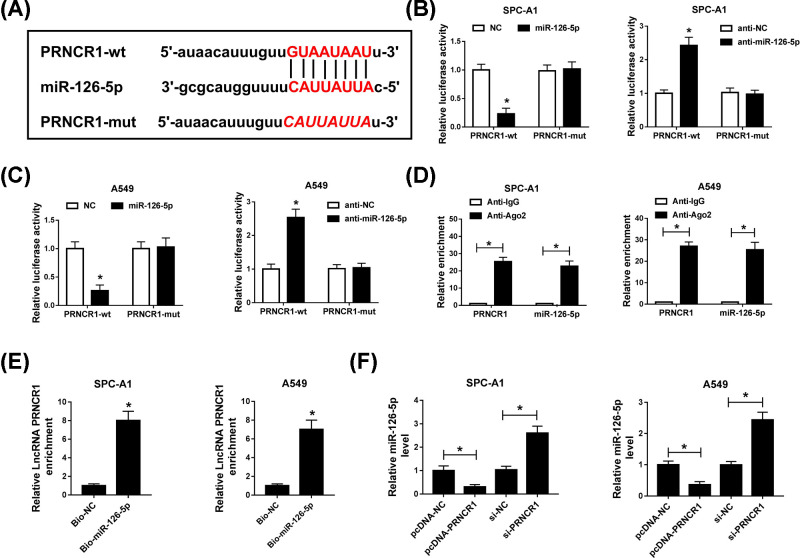
PRNCR1 functioned as a molecular sponge for miR-126-5p (**A**) The interrelation between PRNCR1 and miR-126-5p was predicted by starBase. (**B,C**) Dual-luciferase reporter assay was conducted to identify the impact of miR-126-5p mimic or inhibitor on luciferase intensity controlled by wild-type PRNCR1 or mutant PRNCR1. (**D**) RIP assay was aimed to examine the PRNCR1 and miR-126-5p enrichment in SPC-A1 and A549 cells. (**E**) RNA pull-down assay was carried out to analyze the interaction between PRNCR1 and miR-126-5p in NSCLC cells. (**F**) MiR-126-5p expression was analyzed using qRT-PCR in SPC-A1 and A549 cells. *P<0.05.

Owing to the opposite expression of PRNCR1 and miR-126-5p in NSCLC tissues, the binding domain between PRNCR1 and miR-126-5p was predicted by starBase, and the results were shown in Figure 3A. Subsequently, dual-luciferase reporter assay explored that miR-126-5p mimic specially blocked the luciferase activity of wild-type group, but its inhibitor accelerated the wild-type PRNCR1 fluorescence intensity, whereas they did not influence the luciferase activity in mutant PRNCR1 group (Figure 3B,C). To verify these results, RIP and RNA pull down assays were performed to investigate the interaction between miR-126-5p and PRNCR1. The results exhibited that PRNCR1 and miR-126-5p co-precipitated with pulled down Ago2 in RIP assay, and RIP assay results of that PRNCR1 was up-regulated in introducing Bio-miR-126-5p group, which were indicative of miR-126-5p was a target gene of PRNCR1 (Figure 3D,E). Furthermore, miR-126-5p level was regulated by PRNCR1, it was efficiently decreased by overexpression of PRNCR1, and significantly augmented by PRNCR1 silencing in SPC-A1 and A549 cells (Figure 3F). In short, the evidence displayed that miR-126 was targeted by PRNCR1.

### PRNCR1 regulated cell proliferation, apoptosis, migration, invasion, and EMT via sponging miR-126-5p in NSCLC

**Figure 4:**
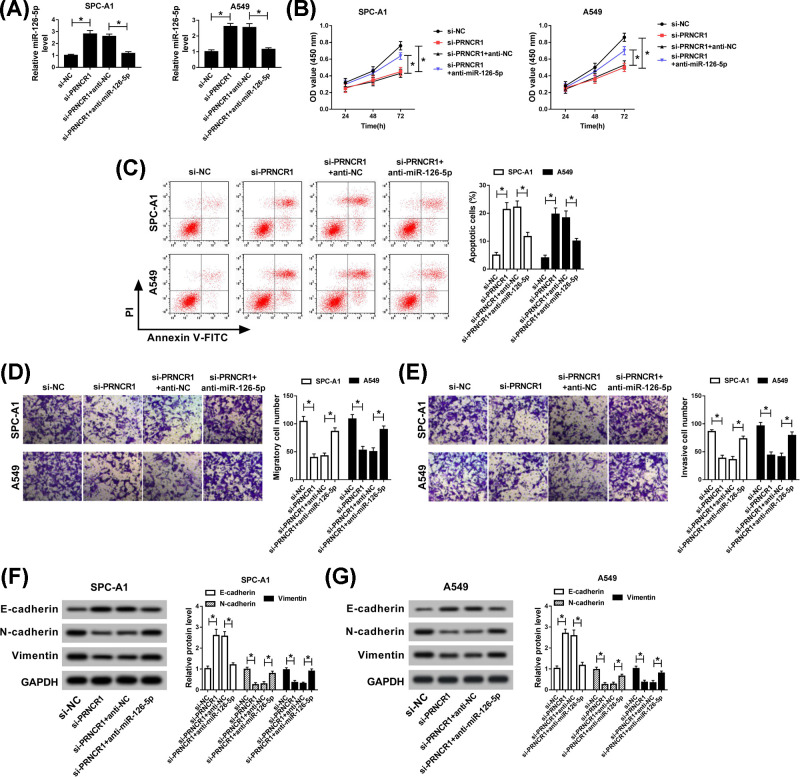
PRNCR1 regulated NSCLC cells proliferation, apoptosis, migration, invasion, and EMT via sponging miR-126-5p Si-NC, si-PRNCR1, si-PRNCR1+anti-NC, or si-PRNCR1+anti-miR-126-5p was introduced into SPC-A1 and A549 cells, respectively. (**A**) The expression level of miR-126-5p was estimated by qRT-PCR analysis. (**B**) CCK-8 assay was employed to evaluate the proliferation of SPC-A1 and A549 cells. (**C**) Apoptotic cells were analyzed utilizing flow cytometry. (**D,E**) Transwell assay was applied to identify SPC-A1 and A549 cells migration and invasion *in vitro*. (**F,G**) EMT level was evaluated via measuring expression levels of E-cadherin, N-cadherin, and Vimentin using Western blot assay. *P<0.05.

According to the above description, the molecular mechanism between miR-126-5p and PRNCR1 was researched subsequently. First, si-NC, si-PRNCR1, si-PRNCR1+anti-NC, or si-PRNCR1+anti-miR-126-5p was transfected into SPC-A1 and A549 cells, and miR-126-5p level, which was expedited by PRNCR1 knockdown, was relieved via introducing miR-126-5p inhibitor (Figure 4A). Simultaneously, after incubation for 72 h, the effect of PRNCR1 deletion on SPC-A1 and A549 cells proliferation was rescued by miR-126-5p inhibitor (Figure 4B). In addition, cell apoptotic rate was determined utilizing flow cytometry, and the results showed that the promotion effect of PRNCR1 down-regulation on cell apoptosis was restored via transfecting miR-126-5p inhibitor in SPC-A1 and A549 cells (Figure 4C). The results of transwell assay uncovered that miR-126-5p inhibitor abrogated the repression impacts of PRNCR1 silencing on cell migration and invasion abilities *in vitro* (Figure 4D,E). Moreover, the capacity of EMT was indicated by the expression of E-cadherin, N-cadherin, and Vimentin, and the results demonstrated that the inhibitory effect of PRNCR1 knockdown on EMT was recuperated by miR-126-5p inhibitor (Figure 4F,G). These findings meant that the role of PRNCR1 deletion in cell behaviors and EMT was abolished by miR-126-5p inhibitor in SPC-A1 and S549 cells.

### MTDH was a target gene of miR-126-5p

**Figure 5:**
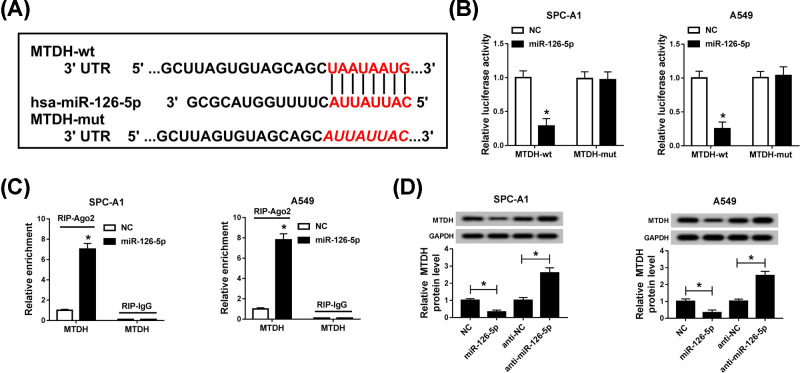
MTDH was a target gene of miR-126-5p (**A**) Targetscan prediction of binding domain between miR-126-5p and MTDH was exhibited. (**B,C**) The interrelation between miR-126-5p and MTDH was analyzed by (**B**) dual-luciferase reporter assay and (**C**) RIP assay. (**D**) MTDH expression level was detected using Western blot assay in SPC-A1 and A549 cells. *P<0.05.

Previously, we verified the binding sites between miR-126-5p and PRNCR1. Then, the downstream genes of miR-126-5p were searched by Targetscan, and we found that MTDH might be one of the targets of miR-126-5p (Figure 5A). The results of the dual-luciferase reporter assay exhibited that there was a targeting interaction between miR-126-5p and MTDH. The miR-126-5p mimic obviously constrained the luciferase activity in wild-type group, while it did not impact binding domain-mutant MTDH expression (Figure 5B). Furthermore, RIP assay results revealed that miR-126-5p and MTDH were co-precipitated with Ago2 pulled down (Figure 5C). Subsequently, we treated SPC-A1 and A549 cells with miR-126-5p mimic or inhibitor, the mimic of miR-126-5p hampered MTDH expression, which was contrasted to the effect of miR-126-5p inhibitor on MTDH protein expression *in vitro* (Figure 5D). From the above descriptions, we emerged that MTDH was a downstream gene of miR-126-5p.

### MiR-126-5p modified cell proliferation, apoptosis, migration, invasion, and EMT by targeting MTDH

**Figure 6:**
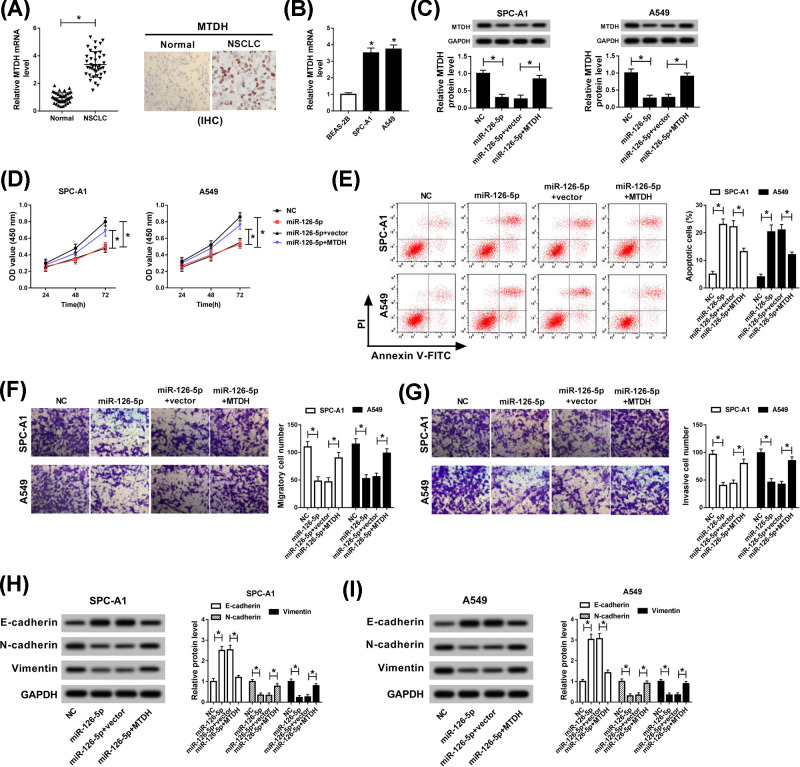
miR-126-5p modified cell proliferation, apoptosis, migration, invasion, and EMT via targeting MTDH (**A,B**) Level of MTDH in NSCLC tissues and cell lines was detected by qRT-PCR. SPC-A1 and A549 cells were transfected with NC, miR-126-5p, miR-126-5p+vector, or miR-126-5p+MTDH, (**C**) and MTDH expression level was examined by Western blot. (**D**) The proliferation abilities of SPC-A1 and A549 cells were analyzed by CCK-8 assay. (**E**) Apoptotic cells were analyzed using flow cytometry. (**F,G**) Transwell assay was performed to assess cell migration and invasion in NSCLC cells. (**H,I**) The expression levels of EMT-relative proteins, including E-cadherin, N-cadherin, and Vimentin were identified via Western blot assay. *P<0.05.

Because of MTDH has been confirmed as the target gene of miR-126-5p in the above introduction. Next, we explored that the mRNA level of MTDH was apparently elevated in NSCLC tissues and cell lines, and an effective high level of MTDH was observed in NSCLC tissues compared with paired control by means of immunohistochemistry assay (Figure 6A,B). In order to investigate the role of miR-126-5p in mediating the progression of NSCLC by targeting MTDH, NC, miR-126-5p, miR-126-5p+vector, or miR-126-5p+MTDH was transfected into SPC-A1 and A549 cells. The protein expression of MTDH, which was reduced by miR-126-5p, was regained via up-regulating MTDH in SPC-A1 and A549 cells (Figure 6C). Additionally, the capacity of cell proliferation was assessed via CCK-8 assay, and the results discovered that MTDH reversed the inhibitory effect of miR-126-5p on NSCLC cell proliferation (Figure 6D). Apoptotic cells were analyzed by flow cytometry, and we found that the acceleration role of miR-126-5p mimic in cell apoptosis was overturned by overexpression of MTDH in SPC-A1 and A549 cells (Figure 6E). Moreover, the inhibitory impacts of miR-126-5p mimic on the capacities of cell migration and invasion were both reverted via introducing MTDH (Figure 6F,G). Lastly, we investigated the regulatory roles of miR-126-5p and MTDH in EMT, the results manifested MTDH could abrogate the repression effect of miR-125-5p mimic on EMT *in vitro* (Figure 6H,I). These data indicated that miR-126-5p modified cell behaviors and EMT via targeting MTDH in NSCLC cells.

### PRNCR1 impacted on the progression of NSCLC via miR-126-5p/MTDH axis

**Figure 7:**
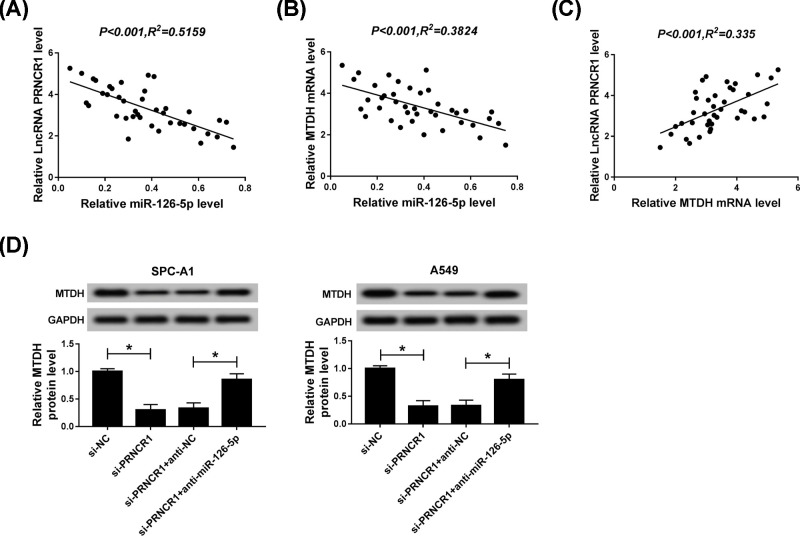
PRNCR1 impacted on the progression of NSCLC via miR-126-5p/MTDH axis (**A**) QRT-PCR was used to analyze the correlation between miR-126-5p and PRNCR1. (**B**) The correlation between miR-126-5p and MTDH was identified using correlation analysis. (**C**) QRT-PCR was employed to verify the relationship between MTDH and PRNCR1. (**D**) After transfection with si-NC, si-PRNCR1, si-PRNCR1+anti-NC, si-PRNCR1+anti-miR-126-5p, the protein expression of MTDH was evaluated utilizing Western blot. *P<0.05.

The assay focused on the correlation among PRNCR1, miR-126-5p, and MTDH. To understand the interrelations among them, we conducted correlation analyses of the expression of PRNCR1, miR-126-5p, and MTDH in 40 NSCLC tissues and matched normal tissues. The analyses results uncovered that miR-126-5p was negatively correlated with PRNCR1 and MTDH (Figure 7A,B), while PRNCR1 was passively related to MTDH (Figure 7C). In the end point, the expression of MTDH was detected by Western blot assay after transfection with si-NC, si-PRNCR1, si-PRNCR1+anti-NC, si-PRNCR1+anti-miR-126-5p into SPC-A1 and A549 cells, the results exhibited that PRNCR1 silencing strikingly retarded MTDH expression, whereas this repression role was relieved by miR-126-5p inhibitor (Figure 7D). The evidence implied that PRNCR1 modulated the development of NSCLC through miR-126-5p/MTDH axis.

## Discussion

In the present study, we discovered that PRNCR1 was up-regulated in NSCLC tissues and cell lines. Recently, accumulating documents have revealed that ncRNAs contributed to the epigenetic regulation in tumors [22]. According to ncRNAs length, they were divided into two types, lncRNAs and miRNAs, respectively. As we all know, lncRNAs are crucial participation factors in multiple malignant cancers [23–25]. Simultaneously, increasing evidence recorded the biological role of lncRNAs in the pathogenesis of lung cancer [26,27].

Moreover, lncRNAs are related to many aspects of the cancer process, including cancer initiation and metastasis [28]. Increasing researches have emerged that PRNCR1 could facilitate various human tumors, such as prostate cancer [29] and colorectal cancer [12]. In our study, the overexpression of PRNCR1 in NSCLC tissues and cell lines (SPC-A1 and A549) was in the light of the previous study [13]. This research also showed that PRNCR1 silencing could enhance cell apoptosis, block proliferation, migration, invasion, and EMT, of which EMT served an essential role in cancer progression [30]. These results meant that PRNCR1 was an oncogene in NSCLC. Based on previous records, lncRNAs exert their functions by interacting with miRNAs. Herein, starBase software was performed to predict the interrelation between PRNCR1 and miRNAs. In accordance with the results, we found that miR-126-5p was one of the predicting target genes of PRNCR1.

Over the past decades, miRNAs play a vital role in cell proliferation, apoptosis, and other cellular processes of tumors [31]. Researches have emerged that miR-126 repressed lung cancer cells migration and invasion [32]. EMT, which can be characterized as a loss of cell adhesion, along with the gain of cell migration, plays a critical function in the early progression of cancer cell metastasis. Additionally, miR-126 has been recorded to suppress cell proliferation in lung cancer [33,34]. Interestingly, miR-126 contains two miRNA chains, including miR-126-3p and miR-126-5p [35]. Shibayama et al. [36] reported that miR-126-5p up-regulation was involved in cytarabine resistance and caused poor prognosis of acute myeloid leukemia patients. Furthermore, miR-126-5p was also proved to act as a tumor suppressor in multiple cancers [17,37], supplying evidence for blocking cancer cell proliferation and invasion. In the present study, the miR-126-5p level was evidently curbed in NSCLC tissues and cell lines, which was in line with the previous clinical researches [38]. In keeping with expectation, miR-126-5p was proved to be the target gene of PRNCR1, and the impacts of PRNCR1 knockdown on cell behaviors and EMT were totally abrogated by miR-126-5p inhibitor in NSCLC cells.

In spite of those findings of miR-126-5p acted as a tumor-inhibiting factor in NSCLC, the elaborate mechanism of miR-126-5p in NSCLC cells is still largely unknown. To investigate the regulatory mechanism of miR-126-5p-mediated pathological roles in NSCLC, online software of Targetscan was employed to analyze the downstream genes. Then, we found that miR-126-5p bound to the 3′-untranslated region (3′-UTR) of MTDH. Furthermore, the ectopic expression of miR-126-5p with special mimic or inhibitor could regulate MTDH protein level. MTDH, which is located at chromosome 8q22.5, is a type of single-pass transmembrane protein, containing 582 amino acids [39]. On the basis of previous evidence, up-regulation of MTDH frequently occurred in multiple cancers, such as melanoma, breast cancer, and prostate cancer and the behavior of MTDH overexpression was also related to poor clinical outcome [40]. From our study, the level of MTDH was highly up-regulated in NSCLC tissues and two cell lines. Especially, overexpression of MTDH rescued the effect of miR-126-5p mimic on cell behaviors and EMT. That was to say, miR-126-5p directly targeted MTDH. Based on this, we regarded that MTDH served as an oncogenic gene in the progression of NSCLC, showing as the promoting effect of MTDH on the aggressive cell behaviors, including cell proliferation, migration, invasion, and EMT in the two NSCLC cells.

In summary, levels of PRNCR1 and MTDH were obviously increased, but miR-126-5p was distinctly decreased in NSCLC tissues and cell lines (SPC-A1 and A549). Furthermore, miR-126-5p was targeted by PRNCR1 and directly targeted MTDH. Functionally, PRNCR1 exerted its roles in cell proliferation, apoptosis, migration, invasion, and EMT via miR-126-5p/MTDH axis in NSCLC cells. However, this research was not all-sided; further studies were needed to reveal the deep regulatory mechanism of PRNCR1, such as animal experiments and the other target genes of PRNCR1 as the objective in further investigations.

## Highlights

1. Levels of PRNCR1 and MTDH were high, while miR-126-5p expression was low in NSCLC tissues and cell lines.
2. miR-126-5p directly targeted MTDH, but was targeted by PRNCR1.
3. Knockdown of PRNCR1 promoted cell apoptosis, impeded proliferation, migration, invasion, and EMT in NSCLC cells.
4. PRNCR1 impacted on the progression of NSCLC via miR-126-5p/MTDH axis.

## Data Availability

The analyzed datasets generated during the present study are available from the corresponding author on reasonable request.

## Abbreviations

BSA: bovine serum albumin
CCK-8: cell counting kit 8
EMT: epithelial–mesenchymal transition
FBS: fetal bovine serum
MALAT1: metastasis-associated lung adenocarcinoma transcript 1
MTDH: metadherin
NC: normal control
NSCLC: non-small cell lung cancer
PFA: paraformaldehyde
PRNCR1: long non-coding RNA prostate cancer non-coding RNA 1
PVDF: polyvinylidene fluoride
qPCR: quantitative real-time polymerase chain reaction
qRT-PCR: quantitative real-time polymerase chain reaction
RIP: RNA immunoprecipitation
RPMI: Roswell Park Memorial Institute
SDS/PAGE: sodium dodecyl sulfate/polyacrylamide gel electrophoresis
TBST 1´: Tris-buffered saline Tween-20
